# Development of anchialine cave habitats and karst subterranean estuaries since the last ice age

**DOI:** 10.1038/s41598-019-48058-8

**Published:** 2019-08-15

**Authors:** Peter J. van Hengstum, Jacque N. Cresswell, Glenn A. Milne, Thomas M. Iliffe

**Affiliations:** 1 Department of Marine Sciences, Texas A&M University at Galveston, Galveston, Texas 77554 USA; 2 Department of Oceanography, Texas A&M University, College Station, Texas 77843 USA; 3 Department of Marine Biology, Texas A&M University at Galveston, Galveston, Texas 77554 USA; 4 Department of Earth and Environmental Sciences, University of Ottawa, Ottawa, Ontario K1N 6N5 Canada

**Keywords:** Ocean sciences, Biogeography, Speciation

## Abstract

Extinction models generally predict that coastal and neritic fauna benefit during sea-level rise (transgression), whereas sea-level retreat (regression) diminishes their suitable habitat area and promotes evolutionary bottlenecks. Sea-level change also impacts terrestrial island biogeography, but it remains a challenge to evidence how sea-level rise impacts aquatic island biogeography, especially in the subterranean realm. Karst subterranean estuaries (KSEs) occur globally on carbonate islands and platforms, and they are populated by globally-dispersed, ancient ecosystems (termed *anchialine*). Anchialine fauna currently exhibit a disjunct biogeography that cannot be completely explained by plate tectonic-imposed vicariance. Here we provide evidence that anchialine ecosystems can experience evolutionary bottlenecks caused by habitat reduction during transgression events. Marine-adapted anchialine fauna benefit from habitat expansion during transgressions, but fresh- and brackish-adapted fauna must emigrate, evolve to accommodate local habitat changes, or are regionally eliminated. Phanerozoic transgressions relative to long-term changes in subsidence and relief of regional lithology must be considered for explaining biogeography, evolution, local extirpation or complete extinction of anchialine fauna. Despite the omission of this entire category of environments and animals in climate change risk assessments, the results indicate that anchialine fauna on low-lying islands and platforms that depend upon meteoric groundwater are vulnerable to habitat changes caused by 21^st^ century sea-level rise.

## Introduction

Sea-level oscillations during the last 500 million years (Phanerozic Eon) have impacted marine and terrestrial island biogeography and evolution by modifying habitat availability and opportunities for organismal gene flow^1–3^. It is generally thought that sea-level regressions can reduce the areal extent of coastal and neritic habitats and can cause bottlenecks in the marine realm^4–6^, whereas terrestrial island fauna and flora benefit from habitat expansion during regressions^7–10^. There is an elevated risk of coastal zone defaunation during the Anthropocene from several human-caused factors like habitat degradation and urbanization^11^, but disentangling how modest rates of current sea-level rise threatens aquatic island fauna remains difficult to assess^12,13^.

**Figure 1:**
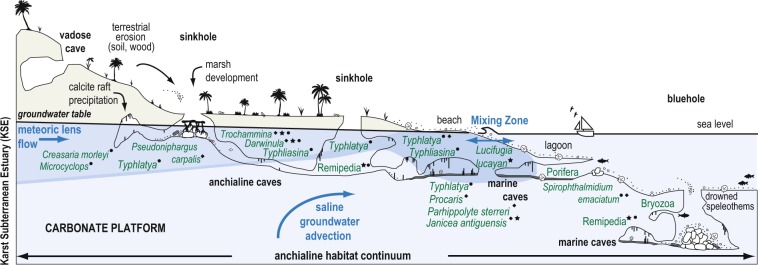
Conceptual model of a karst subterranean estuary and the anchialine habitat continuum created by subsurface groundwater variability. Western North Atlantic anchialine fauna (e.g., fish, shrimps, decapods, ostracodes, foraminifera, marine Porifera and Bryozoa) are positioned in their typical habitat in the karst subterranean estuary. Since most caves do not typically poses all potential sub-habitats, this model is based on observations from several localities, including. The Bahamas (★), Yucatan Peninsula in Mexico (●), and Bermuda (♦).

Worldwide on carbonate islands and platforms, subsurface mixing of rain and marine water creates karst subterranean estuaries (KSEs, Fig. 1). Hydrographically, subterranean estuaries are analogous to other coastal estuaries by having an upper meteoric water mass of varying salinity buoyed on a saline groundwater mass below^14,15^. These two groundwater bodies often destabilize in the subsurface to create mixing zones^16–19^, and their oceanic discharge impacts global biogeochemical cycles^15,20^. Only in the late 20^th^ century did technical scuba diving procedures allow human exploration of KSEs through flooded caves, which lead to the discovery of their unique ecosystems, fauna, and habitats that are now prefaced with the adjective ‘anchialine’^21^. The fossil record^22–24^ and molecular phylogenetics^25–27^ suggests that anchialine fauna and ecosystems persisted through the Phanerozoic and predate angiosperms, and their evolutionary history and biogeochemical functioning can inform early Paleozoic marine ecosystems and invertebrate evolution^28,29^. Steep environmental gradients create diverse benthic and pelagic sub-habitats in the subsurface from the ocean transecting inland (Fig. 1), such that aquatic coastal caves are often categorized as freshwater caves (meteoric water mass), anchialine caves (both water masses), or marine caves (saline water mass). Despite this segregation, some fauna have a modern distribution and evolutionary history in both water masses (e.g., atyid shrimps^30^, *Hadziidae* amphipods)^31,32^. If these habitats are also linked through allogenic succession, then it is perhaps more appropriate to consider these sub-habitats as part of an anchialine habitat continuum (Fig. 1).

Pioneering cave ecologist Riedl first hypothesized that coastal cave habitats experienced allogenic succession from sea-level forced vertical migration of KSEs, which isolated subterranean aquatic fauna and promoted speciation through vicariance^33^. This process was subsequently termed the ‘regression model’^34,35^, which could be caused by either tectonic uplift or isostatic crustal adjustment, and was used to evaluate the disjunct biogeography of continental and coastal subterranean fauna. However, significant barriers exist for developing physical data on developmental succession in anchialine habitats and KSEs, during either regressions or transgressions. Instrumental records (e.g., decadal scale) of hydrographic change in KSEs are unavailable, and speleothems (e.g., stalagmites) do not document environmental change when caves are flooded^36,37^. Soft-bodied endemic cave fauna have a poor fossil preservation, and picturesque clean cave galleries are created by either poor sedimentation in caves^38^, or subsurface currents blowing-out cave sediment. Thus far, available sediment records of environmental change in KSEs are either temporally-fragmented^39,40^, or only document the hydrographic history of an individual groundwater mass^38,41,42^. These knowledge gaps mean that 21^st^ century marine ecosystem risk assessments have little evidence to support forecasting how sea-level rise will impact global *subterranean* aquatic island fauna.

Here we present the most complete record yet known of developmental succession in anchialine habitats from concomitant relative sea-level rise and vertical migration of a KSE since the last ice age. The highest quality sediment record yet found in an underwater cave in Bermuda documents the turnover of anchialine habitats, and their sub-habitats, in response to vertical migration of the KSE. Sea-level rise during the early Holocene first flooded cave passages with a meteoric lens, followed by a paleo mixing zone, and finally saline groundwater to create modern marine cave habitats in western Bermuda. Developmental succession of anchialine habitats during a transgression is now resolved, and more significantly, the results illuminate how sea-level rise can force subsurface aquatic island fauna to experience bottleneck events. It is highly likely that this process impacted the evolutionary history of global subsurface aquatic island fauna during the Phanerozoic. More problematically, 21^st^ century island-based marine ecosystem risk assessments are incomplete if the impact of sea-level rise on anchialine ecosystems is not regionally evaluated.

## Study Site

Bermuda is a ~35 million year old volcanic seamount in the North Atlantic Ocean capped by Quaternary-aged carbonates (limestone) that were deposited during sea-level highstands, interspaced by paleosols that accumulated during the ice ages^43^. The limestone units are lithified wind-blown dunes of shallow marine carbonate particles^44^, which subsequently weathered into a mature karst landscape. Large caves in Bermuda were first dissolved by both rain and groundwater, which then experienced repetitive ceiling collapse events^45–47^. There is no location in Bermuda’s underwater caves where the volcanic-limestone contact is currently exposed.

Bermuda is an ideal location for this study because the flooded caves are an established biodiversity hot spot of endemic anchialine fauna, and the region has served as a model for understanding the evolutionary history of anchialine fauna^48^. The ~30–80 m thick carbonate cap and its caves^49,50^ would have been dry during the last glacial maximum (~20,00 years ago) when local relative sea level and groundwater levels were ~120 m lower^51^. On the Bermuda carbonate platform, deglacial sea-level and groundwater-level rise first flooded topographic depressions to create freshwater lakes, which subsequently converted to marine carbonate lagoons with complete platform flooding^52,53^. Modern Bermudian flooded caves must have similarly developed by the upward displacement of the KSE, with endemic anchialine fauna migrating into newly created ecospace either from the volcanic lithology below or through oceanic dispersal from elsewhere^48^. Sediment has accumulated on Bermuda’s cave floors from minimal groundwater current velocities, and most caves in western Bermuda are at the final stage of oceanic flooding with active modern groundwater-seawater circulation.

**Figure 2:**
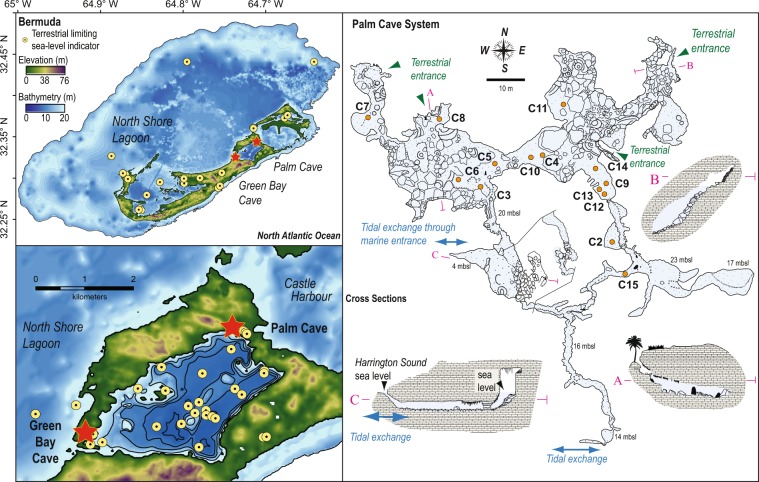
Palm Cave System in Bermuda. (**a**) Digital elevation model of Bermuda^93^ in the North Atlantic Ocean with (**b**) detailed map of Harrington Sound^52^ plotted in ArcGIS. Yellow markers identify locations of geological sea-level indicators from Bermuda (peat, *n = *113, Supplementary Dataset). (**c**) Detailed survey of Palm Cave (adapted after original survey of Jason Richards), with locations of sediment cores and cave entrances.

**Figure 3:**
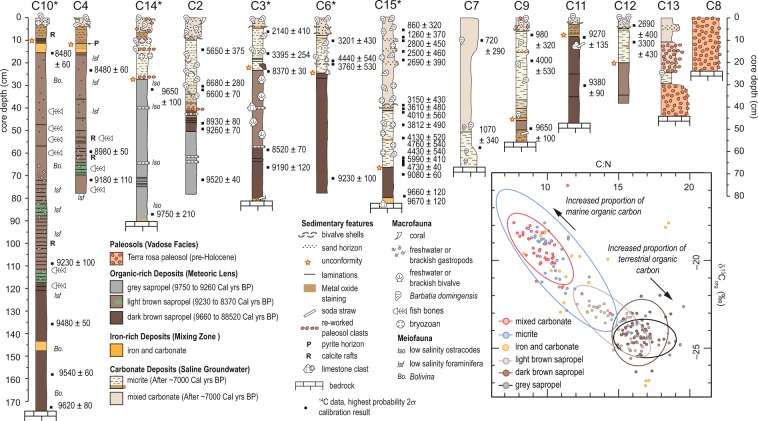
Preserved sedimentary and biological archives. The sediment and biological remains deposited into Palm Cave over the last 10,000 years. Inset is crossplot of δ^13^C_org_ and C:N values on sub-samples from cores marked with an asterisk to evaluate the relative contribution of terrestrial versus marine organic carbon to the sedimentary record, with 95% ellipse confidence intervals around the mean values for individual sedimentary units (except iron-rich deposits).

On the northeastern margin of Harrington Sound in Bermuda, the Palm Cave System occurs to a maximum depth in the subsurface of 23 m below modern sea level (mbsl). There is no halocline in Palm Cave because a meteoric lens does not develop on the narrow isthmus between Harrington Sound and Castle Harbour, so the passages are all flooded by oxygenated saline groundwater (Fig. 2). Despite the absence of a local meteoric lens, Palm Cave is part of the anchialine habitat continuum, and can be colloquially referred to as a marine cave (Fig. 1). In summer 2015, the conditions of the saline groundwater mirrored the impact of strong summertime evaporative and radiative forcing on the adjacent Harrington Sound source water (pH of 7.8 ± 0.2, 28.5 ± 0.2 °C, 38.7 ± 0.4 psu, Fig. 2). Multiple physical openings from the coastal lagoon (i.e., Harrington Sound) and subaerial forest landscape (e.g., 32.34°, −64.71) allow terrestrial and marine organic matter to erode into the cave. Sediment push cores (*n* = 13) were collected using technical cave diving procedures (Fig. 2, Supplementary Table S1) from the deepest parts of the cave that preserve sediment accumulations, and areas with representative sedimentary units. Multiple cores sampled the stratigraphy to limestone bedrock (Fig. 3).

## Sedimentaray Deposits and Habitats

The deposits from Palm Cave can be organized into four groups (paleosols, organic-rich deposits, iron-rich deposits, and carbonate deposits), and further subdivided into seven units (Fig. 3). These units correspond to hydrographic and environmental change in the cave from internal and external flooding of the Bermuda carbonate platform by concomitant groundwater and relative sea-level rise (Fig. 2). Limitations of the record include decreased sedimentation rates from ~8500 to ~7000 calibrated years before present (Cal yrs BP), and no one single location in the cave preserves a complete Holocene environmental history. However, the recovered deposits collectively provide the most detailed physical and biological picture yet known of Holocene environmental change in a KSE, and are described below from oldest to youngest.

### Pre-Holocene vadose deposits

The oldest deposits are pre-Holocene (>11,600 years ago) terra rosa paleosols, which occur at the base of cores (core 13), in areas with negligible Holocene sedimentation (core 8), and near terrestrial openings. These coarser-grained sediments have a deep red color (Supplementary Fig. S1, Table S2 and Fig. S2), and they contain no fossil material (Fig. 3). Mineralogically, they are primarily crandallite (mean 52.6%), kaolinite (mean 16.5%), quartz (22.8%) and goethite (8.1%, Supplementary Table S4), which is similar to Bermudian Pleistocene-aged terra rosa paleosols^54^. African dust is an important contributor to Bermudian soils during Quaternary Ice Ages^54^, which helps create diagnostic terra rosa soils that are known to erode into Bermudian caves^55^. Similarly, paleosol eroded into Palm Cave prior to Holocene flooding.

### Freshwater habitats in a meteoric lens

The preserved sedimentary and biological remains (e.g., fish bones, foraminifera, ostracodes) indicate that freshwater aquatic habitats were created in the Palm Cave from 9750 ± 210 (core 14) to 8370 ± 30 (core 3) Cal yrs BP when a fresh to oligohaline meteoric lens first flooded the cave. The modern marine anchialine ecosystem in Palm Cave could not have colonized these conditions. In contrast, these early Holocene freshwater conditions would have been suitable to Bermuda’s olighaline-adapted anchialine amphipods like *Pseudoniphargus carpalis* and *P. grandimanus*^56^. In eastern passages, grey sapropel (mean 16% organic matter) with calcium-rich layers was accumulating by ~9750 Cal yrs BP (cores 14 and 2, Supplementary Table S3). The organic carbon was primarily terrestrial in origin, based on the stable carbon isotopic content of the bulk organic matter (δ^13^C_org_: −24.1 ± 0.6‰) and relative amounts of organic carbon and nitrogen (C:N ratios: 17.2 ± 1.1). The only meiofauna preserved in the grey sapropel were the benthic ostracodes *Darwinula stevensoni* and *Cypridopsis vidua*, which occur in global freshwater habitats and Yucatan freshwater caves. At ~9620 Cal yrs BP, the deepest cave areas (e.g., cores 4, 10) began accumulating dark brown sapropel (mean 31% organic matter, −24.1 ± 0.9‰, 16.8 ± 1.1, *n* = 43), which pass upcore into a lighter-hued light brown sapropel by ~9230 Cal yrs BP (mean 24% organic matter, δ^13^C_org_: −23.5 ± 0.9‰, C:N: 14.9 ± 1.1, *n* = 39). The timing of the transion between light and dark brown sapropel varied slightly between the core sites, and likely reflect site-specific process impacting sedimentation at these different parts of the cave^41^. Biologically, the sapropel contains benthic foraminiferal assemblages that are dominated by *Polyscammina iophalina*, *Entzia macrescens*, *Tiphotrocha comprimata* (Supplementary Figs S2, S3). At the base of some cores (core 10), *Bolivina* sp. was dominant at first, but the high sedimentation rate at this site indicates these assemblages rapidly transitioned to *Entzia*-dominated assemblages. In modern settings, these benthic foraminifera dominate subtidal anchialine habitats that are flooded by a low salinity (oligohaline) meteoric lens on the Yucatan Peninsula (*Tiphotrocha*, *Entzia*)^42,57^, and subtidal marine settings in Bermuda dominated by terrestrial organic carbon (*Bolivina*)^58^. Calcite rafts occurred intermittently in the organic-rich deposits, which only form near freshwater-air interfaces in caves^40,59^, and their occurrence indicates the continual cave passage flooding. Previous work indicates that the shoreline of an early Holocene inland brackish pond in Harrington Sound was very close to Palm Cave by ~9500 years ago^52^, which likely provided a source of organic-rich sediment that was transported into the cave through a southern tunnel connecting Palm Cave to Harrington Sound (Fig. 2). Continual water-level rise in the cave or conduit collapse events likely decreased organic matter sedimentation at the core sites, as has been observed in Yucatan flooded caves^38^.

### Mixing zone iron curtain deposits

**Figure 4:**
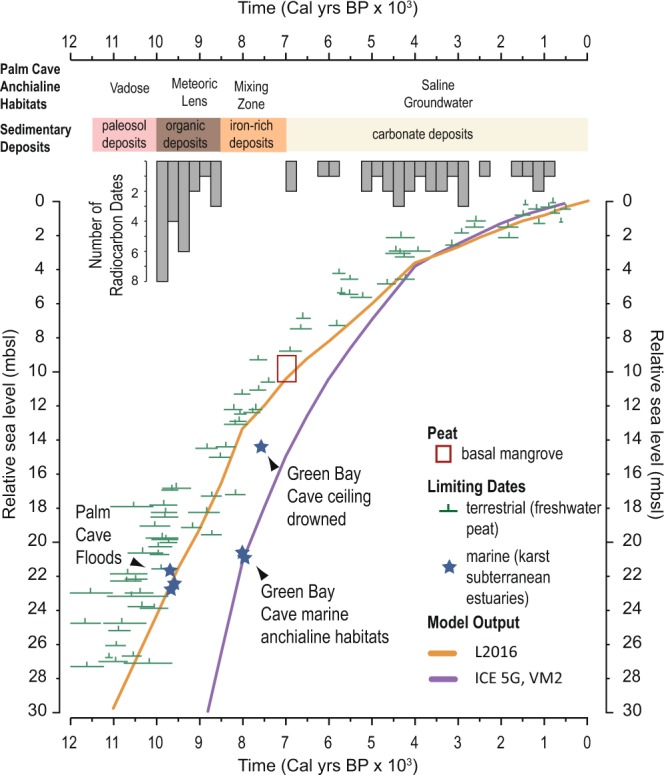
Evidence for Holocene sea-level rise in Bermuda and the allogenic succession of anchialine habitats in Palm Cave, which is caused by the upward migration of the local karst subterranean estuary. Marine limiting sea level indicators from Palm Cave (this study) and Green Bay Cave^40^ (symbol size exceeds age and depth uncertainties), along with a new regional database of terrestrial limiting sea level indicators (freshwater peat, see methods) are compared to the output from two glacial isostatic adjustment models (purple^91^ and orange^68^ lines; see Methods and Supplementary Dataset). The L2016^68^ model allows for marine limiting sea level indicators from Palm Cave and Green Bay Cave to be flooded, while coeval terrestrial limiting dates remain exposed.

The Iron-rich carbonate deposits provide evidence for benthic habitats becoming flooded with saline groundwater, whereby upwelling anoxic saline groundwater was mixing with the overlying freshwater in a paleo mixing zone [base of cores 3, 15 and 9, and intercalated within cores 4,10, 11 and 9 (Fig. 3)]. These deposits were not recovered from shallower sampling locales (Supplementary Table S1). Unlike the terra rosa paleosols, these iron-rich carbonate sediments have a distinctive orange-hue (Supplementary Fig. S1), have a fine texture, and they contain rare marine benthic foraminifera adapted to low-oxic environments (i.e., *Bolivina* spp.). The dominant minerals present were carbonates (mean 45% calcite and aragonite), quartz (mean 27.6%), Fe-based minerals (mean 13.4% goethite, woodhouseite, and lepidocrocite), and less crandallite and kaolinite than the paleosol deposits (Supplementary Table S4). Organic carbon was dominated by marine sources (δ^13^C_org_: −21.2 ± 2.9‰, CN: 13.3 ± 2.9, *n* = 22, Fig. 2). The meiofuna and mineralogy differentiates these sediments from known Fe-oxide deposits produced by microbialites in anoxic saline groundwater^60^. A Saharan dust origin is also not likely, given the contemporaneously flooded cave would have hampered wind-borne dust accumulation (Fig. 4), and African dust export to the western tropical North Atlantic was diminished from ~11,000 to 5,000 years ago^61^.

Alternatively, these iron-rich deposits developed at the deepest elevations from the oxidative precipitation of Fe(II) in an ‘iron curtain’ at the sediment-water interface. On siliciclastic coastlines, the oxidative precipitation of dissolved Fe(II) from the mixing of seawater and groundwater generates a distinctive increase in iron oxide deposits in a subsurface zone^62^. This process is driven by pH gradients between the anoxic saline versus oxygenated freshwater above^63^, and the iron curtain can spatially migrate in response to sea-level^64^ or rainfall^65^ changes. It is likely that anoxic saline groundwater was displaced upwards under sea-level forcing, and iron oxide precipitated when anoxic water upwelled and mixed with an overlaying oxygenated water mass. The previously observed iron-oxide coatings on early Holocene calcite rafts in another Bermudian flooded cave likely formed through the same process^40^.

### Oxygenated marine habitats in saline groundwater

 By ~7000 years ago, the entirety of Palm Cave became a fully-oxygenated marine aquatic habitat, based on radiocarbon dates from core 2 (6680 ± 280, and 6600 ± 70 Cal yrs BP). This is demarcated by widespread carbonate deposition (fine-grained micrite and mixed carbonate), and the appearance of marine pelycopods (e.g., *Barbatia domingensis*), bryozoans (*Cheilostomata*), coral (*Coenocyathus goreaui*), brachiopods and marine ostracodes. The marine foraminifer *Spirophthalmidium emaciatum* also colonizes Palm Cave, which lives in marine caves in Bermuda and Cozumel with oxygenated saline groundwater^66^ (Supplementary Figs S2, S3**)**. White- to brownish-hued micrite deposits often transition into mixed carbonate facies (e.g., cores 14, 2, 15) towards the top of cores, but the timing is site specific (e.g., core 12: ~2690 Cal yrs BP vs. core 15: ~3610 Cal yrs BP). The spatial and temporal variability of carbonate sedimentary units is perhaps related to conduit-specific physical or hydrodynamic processes (e.g., diffuse- vs. conduit-driven saline groundwater circulation). The carbonate deposits contained the highest proportion of marine organic matter based on the C:N and δ^13^C_org_ values (micrite: δ^13^C_org_ −20.4 ± 1.9‰; C:N 11.7 ± 2.0‰, *n* = 40, mixed carbonate facies: δ^13^C_org_ −19.4 ± 1.2‰; C:N 10.3 ± 1.2, *n* = 38). Occasionally, clasts of the terra rosa paleosol become eroded and re-worked into the carbonate deposits (cores 13 and 14). These deposits indicate that conditions in Palm Cave were finally suitable for colonization by the soft-bodied marine anchialine fauna, such as *Parhippolyte sterreri* and *Procaris chacei* sp.

## Sea-Level Forcing of Successional Development

There is striking congruency between when aquatic ecosystems were emplaced in Palm Cave, subaerial indicators of sea-level change from the Bermuda carbonate platform, evidence for carbonate banktop oceanographic changes, and numerical models of relative sea-level rise (Fig. 4). Terrestrial and freshwater peat deposits recovered in contact with the carbonate platform from Bermuda provide *maximum* sea-level indicators (Fig. 4), which constrain glacioisotatic processes and past sea levels^67^. In contrast, cave sedimentary deposits are *minimum* sea-level indicators because groundwater must have achieved this elevation for aquatic habitats develop^58^. The sedimentary and meiofaunal remains in contact with the limestone indicates a meteoric lens flooded Palm Cave by 9750 ± 210 Cal yrs BP at core 14 (21.6 ± 0.3 mbsl), 9620 ± 80 Cal yrs BP at core 10 (22.5 ± 0.3 mbsl), and 9670 ± 120 Cal yrs BP at core 15 (22.7 ± 0.3 mbsl). A comparison with output from the ICE-5G model is within uncertainties of early Holocene minimum (cave-based) and maximum (terrestrial peat) sea-level indicators (Fig. 4, L2016^68^ Model). From previous work in Green Bay Cave in Bermuda, there was a delayed onset in sedimentation to first document aquatic conditions at ~7,900 Cal yrs BP^40^. However, the estimated time for drowning of the ceiling in Green Bay Cave is very close to the anticipated position of relative sea level (Fig. 4, L2016^68^ Model). By 7,000 years ago, both Harrington Sound^52^ and North Lagoon^69^ transitioned into marine carbonate lagoons from continual inundation of the Bermuda carbonate platform by Holocene sea-level rise. This allowed tidal exchange of seawater between the saline groundwater and adjacent marine carbonate lagoons, which initiated oxygenated saline groundwater habitats in Palm Cave. These results indicate that relative sea-level change is a principle driver of successional development of anchialine habitats through initial installation of anchialine habitats by from sea-level forced vertical migration of groundwater. Thereafter, sea-level forced changes to banktop oceanographic-groundwater circulation regimes secondarily modified Bermuda’s anchialine habitats into their current environmental state.

## Global Implications

The successional development of anchialine habitats caused by sea-level rise has multiple implications for the biogeography, evolutionary history, and ecosystem functioning across the anchialine habitat continuum. Today, anchialine fauna include taxa that are adapted to habitats and environmental conditions created by specific groundwater masses in the KSE, in addition to taxa that have an evolutionary history in both water masses. For example, Bahamian remipedes remain in their ancestral marine-based habitat, whereas others like the Yucatan decapod *Creaseria morleyi* have become adapted to the low salinity habitat created by the meteoric lens. On geologic timescales, anchialine fauna and ecosystems must vertically migrate upwards (or downwards) with sea-level rise (or fall) with groundwater masses in the KSE to remain within their suitable ecological tolerance ranges. The sedimentary record from Palm Cave indicates that the available anchialine habitat in northeastern Bermuda associated with a meteoric lens (freshwater to brackish salinity) *decreased* with Holocene sea-level rise. This was coincident with regional reduction in the Holocene aerial extent of Bermuda, and ultimate potential for a meteoric lens to form in the antecedent limestone. The possibility of suitable aquatic habitat is first established by the sea level boundary condition, and thereafter, the subsurface hydrography is modified by other known secondary factors, such as changes in coastal circulation^70^, conduit morphology and connectivity to adjacent marine and terrestrial environments^38,40^, or changing rainfall^71–73^.

**Figure 5:**
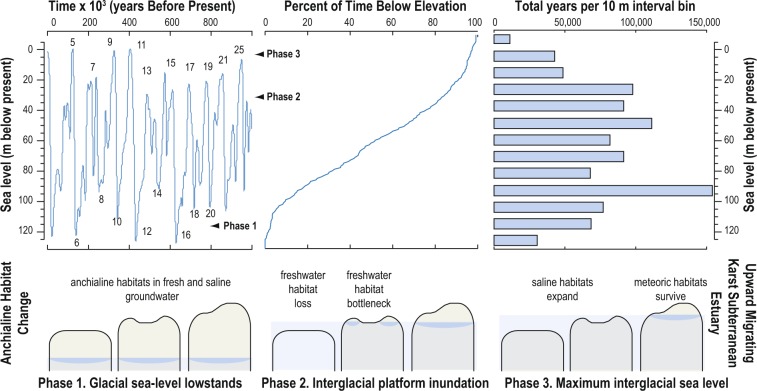
Sea-level change over the last million years impacts anchialine habitat availability. Eustatic sea-level change based on global ice-volume changes^94^, the relative time spent below the modern sea level position, and the time spent at different eustatic sea level elevations spanning the last million years. This is compared to a single transgression event on three idealized carbonate platforms with different topographic relief and vertical migration of their karst subterranean estuaries, which in turn causes bottlenecking (expansion) of the meteoric lens (saline groundwater) anchialine habitats.

On Phanerozoic timescales, transgressions cause bottlenecks to anchialine habitats and fauna dependent upon a meteoric lens, if carbonate platform areal extent and island relief are considered (Fig. 5). The earlier regression model first proposed by Riedl^33,35^ hypothesized how the draining of epicontinental seas on geologic timescales first promoted subsurface habitat colonization. However, the regression model did not evaluate or describe how sea-level oscillations can continuously drive adaptation, community evolution, and potential for regional extinctions. During the Quaternary alone, smaller carbonate platforms and islands were repetitively and completely flooded by sea level during interstadials from the ~100,000-year climate cycle^74^. As such, meteoric lenses contracted, fragmented, and potentially disappeared (e.g., Bermuda, or Cay Sal Bank in The Bahamas). The sediment and preserved meiofauna in Palm Cave clearly documents habitat reduction associated with a contracting meteoric lens in northwestern Bermuda from sea-level rise during the most recent deglaciation. There is little evidence to suggest that this process has not persisted through geologic time, or would have not impacted carbonate platforms elsewhere. This means that fauna and ecosystems in the anchialine habitat continuum that depend upon a meteoric lens must have faced recurrent habitat fragmentation and bottleneck events coincident with sea-level rise linked to the ~100,000-year climate cycle during the Quaternary^74^. Indeed, recently discovered anchialine food web dynamics and habitats that depend on terrestrial dissolved organic carbon and meteoric lenses could not exist on low-relief carbonate islands and platforms during transgressions^17^. The mechanism for carbon and energy transfer between anchialine fauna and ecosystems in saline groundwater devoid of meteoric lenses remains currently unknown. It is likely that global anchialine fauna in the meteoric lens of KSEs experienced increased extinction rates during Pliocene (+6 above present) or late Paleocene (+75 m above present) transgressions^51,75^. Subsurface defaunation on different types of carbonate platforms from transgression-related extinction events requires further evaluation.

In contrast, carbonate platforms with higher elevation may have insulated the anchialine habitat continuum from transgression related bottlenecks during global ice volume, tectonic changes or isostatic changes (e.g., Yucatan Peninsula, Fig. 5). For example, the post-Pliocene migration and diversification history of Yucatan freshwater copepods *Microcyclops* and *Diacyclops* have benefited from the continuous presence of a meteoric lens on the Yucatan Peninsula during the Quaternary, and associated lack of regional bottlenecking during sea-level highstands^76^. Anchialine fauna in the saline groundwater mass of a KSE may actually benefit from habitat expansion during banktop inundation coincident with Quaternary interstadials, potentially favoring increased genetic exchange or speciation through the mixing-isolation-mixing mechanism^77^.

Looking forward, worldwide fauna in the anchialine habitat continuum remain omitted from 21^st^ century marine ecosystem risk assessments^78^. The threat of habitat loss related to subsurface contaminants is now apparent^79^, along with potential impacts from increasing coastal sea surface temperatures^80,81^. However, recent groundwater modeling work indicates that <1 m of sea-level rise can reduce meteoric lens volumes by over 50%, and thus available area of brackish and freshwater ecological niches in the anchialine habitat continuum, when inherited topography and platform flooding are collectively considered^82^. The results presented here indicate that the effects of sea-level rise on the anchialine habitat continuum must also be regionally evaluated. It is likely that anchialine fauna on low-lying carbonate platforms and islands are the most vulnerable to potential bottlenecking during 21^st^ century sea-level rise.

## Methods

After collection (Supplementary Fig. S1 and Table S1), cores were transported back to the laboratory to be split lengthwise for x-radiography, photography, textural and micropaleontological analysis (Supplementary Fig. S2). Cores were sub-sampled at 1-cm intervals downcore for analysis of sedimentary bulk organic matter content using a standard Loss-on-Ignition procedure, whereby the mass lost during combustion at 550 °C for 4.5 hrs is expressed as a weight percent^83^. The textural variability in the coarse sedimentation deposition (e.g., coarse fraction) was quantified in contiguous 1-cm intervals downcore on separate sediment sub-samples using a Sieve-first Loss-on-Ignition procedure^84^. Contiguous 1-cm sediment sub-samples, with a standardized initial volume of 2.5 cm^3^, were first wet sieved over a 63-μm mesh, dried for 12 hours in an oven at 80 °C and weighed to determine the original sediment mass. Samples were the ignited for 4.5 hours at 550 °C in a muffle furnace to remove organic matter from the sediment samples to concentrate the remaining mineral residue (Fig. 5), and re-weighed to determine remaining mineral mass after combustion. The variability in coarse sediment was then expressed as mass per unit volume (D_>63 um_ mg cm^−3^). Core 4 (*n* = 26) and core 10 (*n* = 23) were quantitatively analyzed for preserved subfossil benthic foraminiferal assemblages. Downcore sediment subsamples (2.5 cm^3^, 0.5 cm core width) were sieved over a 63 μm mesh, and wet-picked onto micropaleontological slides for taxonomic identification and assemblage analysis with Q-mode cluster analysis (Supplementary Fig. S4). The qualitative (presence vs. absence) preservation of meiofauna (e.g., ostracodes, foraminifera) and macrofauna (e.g., bivalves, gastropods, Supplementary Fig. S3 and Table S2) in all cores was assess by wet sieving bulk sediment over a 63 μm mesh at 3 to 5 cm downcore, with representative individuals imaged with a Hitachi desktop scanning electron microscope (Supplementary Fig. S4). To further understand the provenance of accumulating bulk organic matter, sediment samples (*n* = 215) from selected cores (3, 6, 10, 14, 15) were analyzed for the stable carbon isotopic value (δ^13^C_org_) and C:N ratio of bulk organic matter. Samples were first treated with a 10% HCl digestion to remove carbonates, followed by geochemical measurements in a Costech 200 Elemental Analyzer connected to Thermo-Electron Delta V Advantage Isotope Ratio Mass Spectrometer. Final δ^13^C_org_ values are reported in per mil notation (‰) relative to the standard Vienna Pee Dee Belemnite (VPDB) for carbon (expressed as parts per mil, ‰), with analytical precision on δ^13^C_org_ better than ± 0.2‰ (1σ) and ± 0.1 on C:N. Age control is provided by radiocarbon dating (*n* = 51, Supplementary Table S3) on a combination of bulk organic matter from the organic-dominated facies, terrestrial plant macrofossils (when available), and marine bivalves. Twenty-seven samples were processed by accelerator mass spectrometry radiocarbon, with twenty-six dated using the Continuous Flow AMS (CFAMS) method. All radiocarbon dates were calibrated to sidereal years before 1950 AD (Cal Yrs BP_1950_) using IntCAL13^85^ (Supplementary Table S3). Samples from notably iron-rich carbonate and paleosols (cores 10 and 4) were subject to X-ray diffraction to determine dominant minerals (Table S4). Selected samples were analyzed on a Bruker-AXS D8 Advanced Bragg-Brentano X-ray powder diffractometer employing the standard XRD laboratory protocols. Final mineral determination was made by comparing the resultant diffractograms with the 2005 International Center for Diffraction Data material identification database to determine final mineralogy (Supplementary Table S4).

A new database of sea-level indicators from Bermuda was compiled from earlier work^52,53,86–89^ that is mostly derived from basal peat in contact with limestone (Supplementary Dataset). This database is currently unavailable in other databases of global sea-level indicators^67^. The radiocarbon-dated sedimentary deposit is these earlier works is often designated simply peat, without any differentiation between brackish and freshwater peat using preserved microfossils (i.e., defined indicative meaning). Nevertheless, much of the new database compiled here is derived from peat collected at the limestone contact^52,89^, and thus can still be conservatively used as maximum sea-level indicators (terrestrial limiting)^67^.

The model results (Fig. 4) were determined using the same glacial isostatic adjustment model as described elsewhere^90^, but using different inputs. These inputs are: (1) a reconstruction of changes in grounded ice distribution and (2) solid Earth viscosity structure. In one case (purple line in Fig. 4), the ice model used was ICE-5G with a viscosity model that provides good fits to a global distribution of various data types (VM291^91^,^92^, with a 90 km thick lithosphere). The other model estimate (orange line in Fig. 4) is also based on the ICE-5G ice history, but with the North American component replaced with output from a calibrated glaciological model (L2016, model #9894^68^). The Earth viscosity model was one found to produce an optimal fit to a regional Holocene sea-level data base that includes the Atlantic and Gulf coasts of the US^68^. The main cause of the difference between the two curves are the higher Earth viscosity values found in L2016^68^, which provides a modeled sea level curve that is most compatible with the Bermuda observations.

## Supplementary information

Supplementary Information

Supplementary Dataset 1
