# Supplementary material for: Development of anchialine cave habitats and karst subterranean estuaries since the last ice age
Source: Sci Rep. 2019 Aug 15;9:11907. doi: 10.1038/s41598-019-48058-8 (PMC6695480; doi:10.1038/s41598-019-48058-8)
Supplement: Supplementary file 1 — Supplementary Information [file 41598_2019_48058_MOESM1_ESM.pdf]

Online Supplementary Information

Development of anchialine cave habitats and karst subterranean estuaries since the last ice age

Peter J. van Hengstum[1,2,*], Jacque N. Cresswell[3], Glenn A. Milne[4], and Thomas M. Iliffe[3]

1. Department of Marine Sciences, Texas A&M University at Galveston, Galveston, Texas, 77554
2. Department of Oceanography, Texas A&M University, College Station, Texas, 77843
3. Department of Marine Biology, Texas A&M University at Galveston, Galveston, Texas, 77554
4. Department of Earth and Environmental Sciences, Ottawa, Ontario, Canada, K1N 6N5

*Corresponding author: vanhenp@tamug.edu

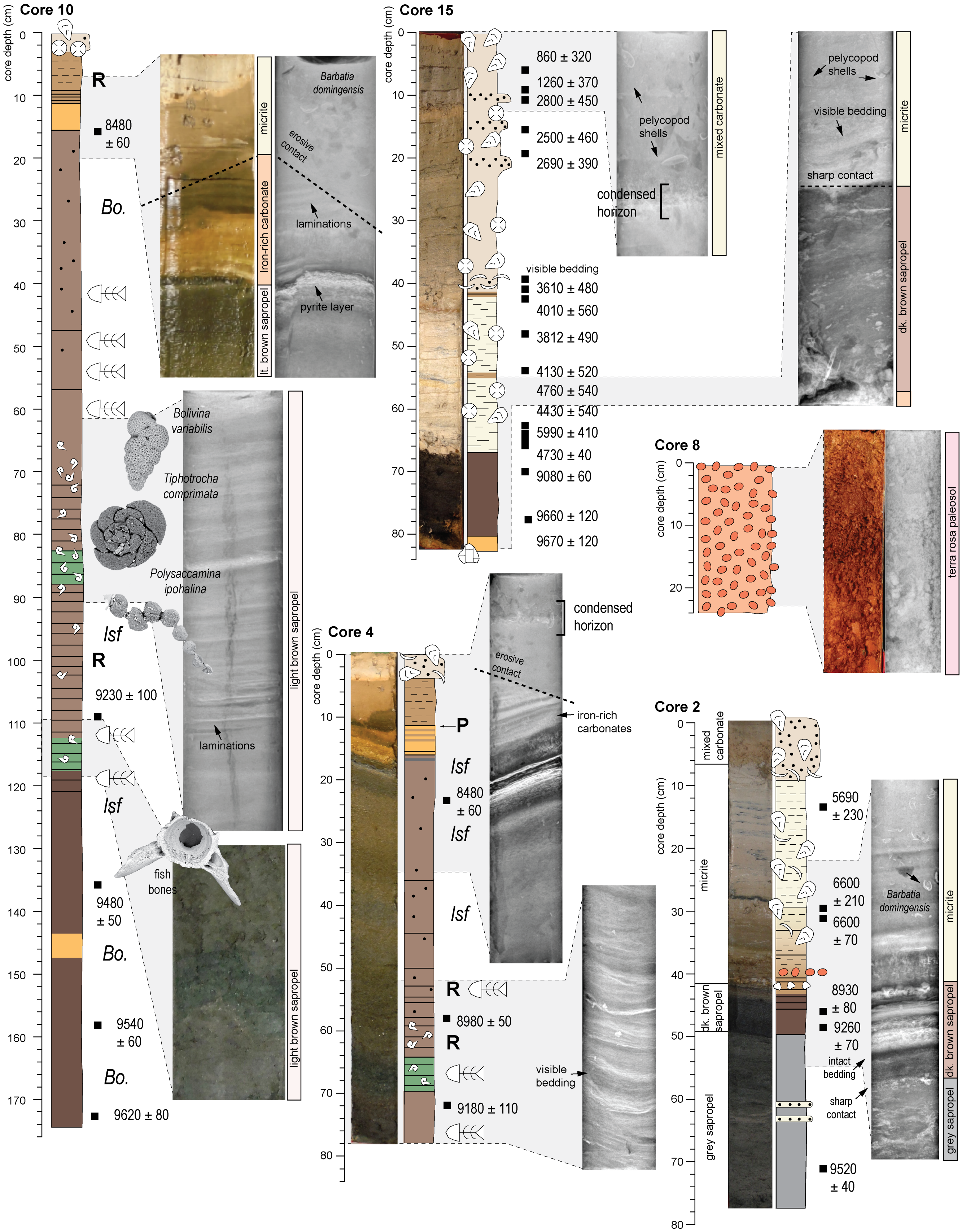

Figure S1| Representative photographs and radiographs for key sedimentary units and transitions, along with the cores logs (and symbols) from the figure in the main text.

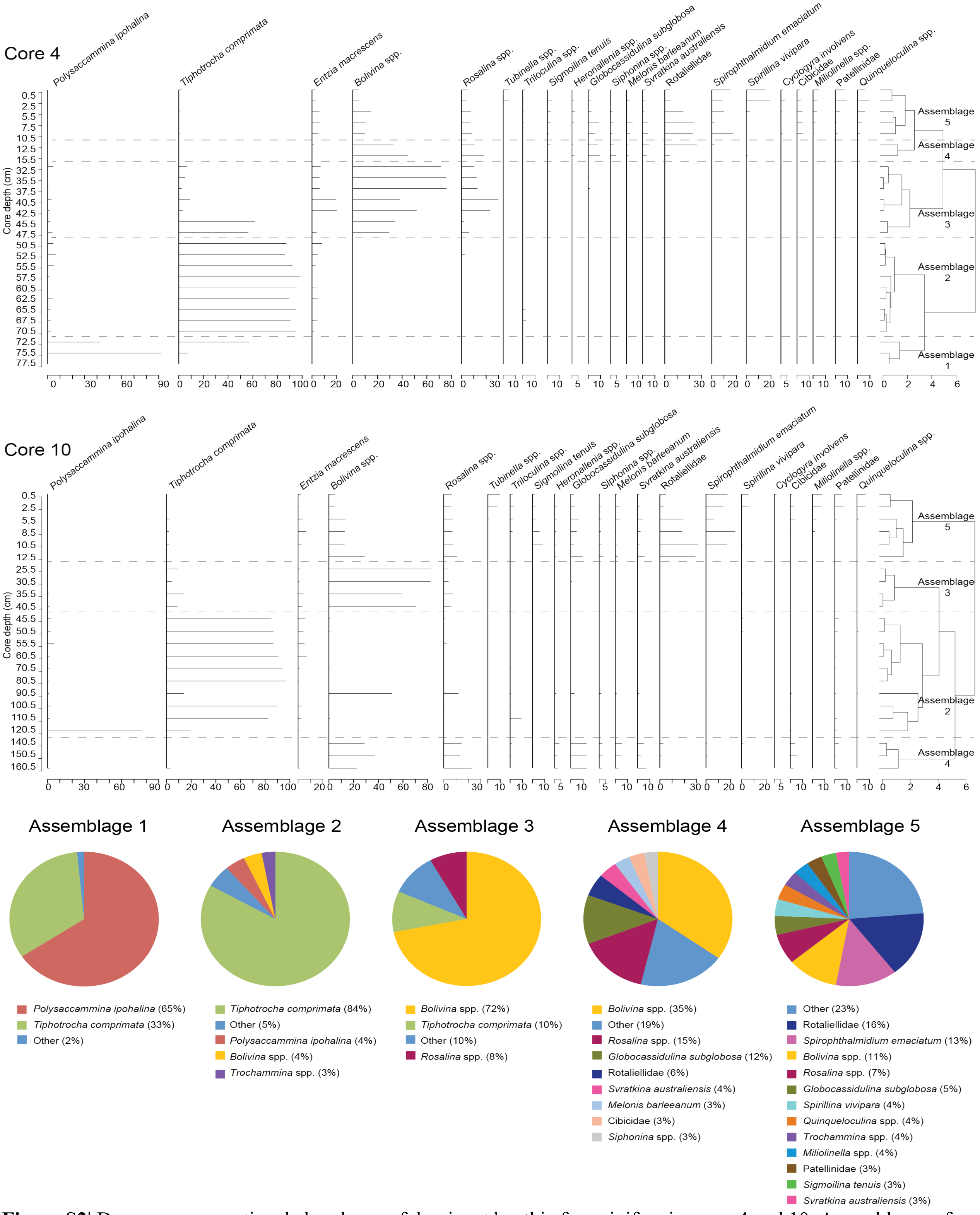

Figure S2| Downcore proportional abundance of dominant benthic foraminifera in cores 4 and 10. Assemblages of foraminifera were identified with Dendrograms produced from Q-mode stratigraphically-constrained cluster analysis on statistically significant taxa[1]. Benthic foraminifera were rare between in core 4 from 15.5 to 32.5 cm, and in core 10 from 12.5 to 25.5 cm, but *Bolivina* sp. was observed.

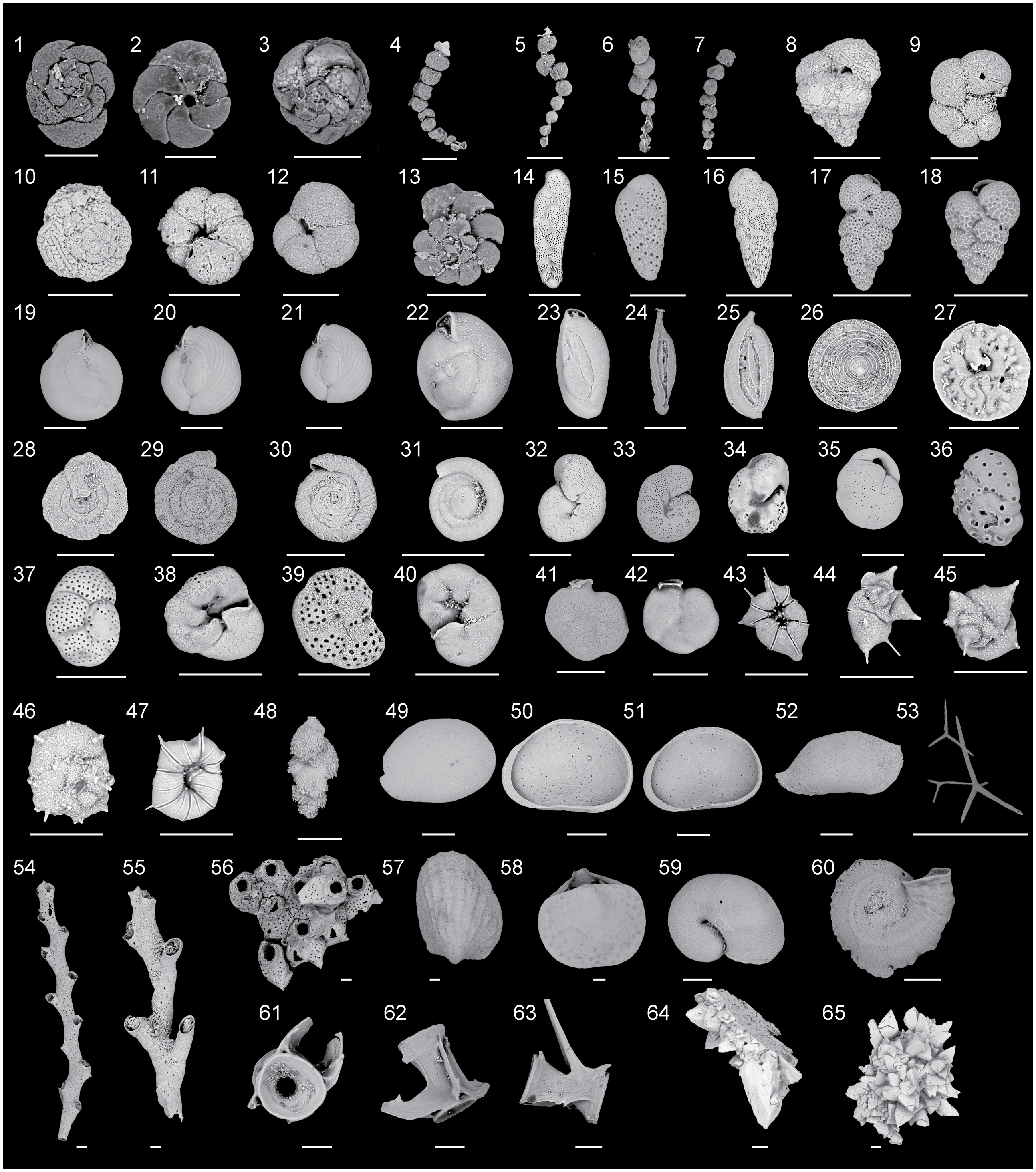

Figure S3| Representative Scanning electron micrographs of significant biological and sedimentary remains preserved in the different sedimentary units from core 4 and 10 (1-48, benthic foraminifera; 49- 65, other). Benthic foraminiferal taxonomy followed published regional[2,3] and international literature[4]. 1-3, Dorsal and ventral views of *Tiphotrocha comprimata* (Cushman & Brönnimann, 1948). 4-7, *Polysaccammina ipohalina* (Scott, 1976). 8, *Eggerella scabra* (Williamson, 1858). 9, *Labrospira evoluta* (Natland, 1938). 10-12, Dorsal and ventral views of *Trochammina quadriloba* (Höglund, 1948). 13, *Entzia macrescens* (Brady, 1870).14, *Bolivina pseudopunctata* Höglund (1947). 15, *Bolivina paula* (Cushman and Ponton, 1932). 16, *Bolivina striatula* (Cushman, 1922). 17-18, *Bolivina variablis* (Williamson, 1858). 19-22, *Miliolinella circularis* (Bornemann, 1855). 23, *Quinqueloculina bosciana* d,Orbigny (1846). 24, *Spirophthalmidium emaciatum* (Haynes, 1973). 25 *Sigmoilina tenuis* (Czjzek, 1848). 26-27, *Patellina corrugata* Williamson (1858). 28, *Mychostommina revertens* (Rhumbler, 1906). 29- 30, *Spirillina vivipara* (Ehrenberg, 1843). 31, *Cyclogyra involvens* (Reuss, 1850). 32-33, *Melonis barleeanum* (Williamson, 1858).

34, *Nonion pauperatum* (Balkwill & Wright, 1885). 35, *Globocassidulina subglobosa* (Brady, 1881). 36, Dorsal view of *Svratkina australiensis* (Chapman, Parr, & Collins, 1934). 37- 40, Dorsal and ventral views of *Rosalina* spp. 41-43, *Siphonina temblorensis* (Garrison, 1959). 43-45, Dorsal and ventral views of *Rotaliella arctica* (Scott & Vilks, 1991). 46-47, Dorsal and ventral views of *Heronallenita* spp. 48, *Trifarina occidentalis* (Cushman, 1922). 49 dorsal view of ostracode *Cypridopsis vidua* (Müller, 1776) 50-51, ventral view of ostracode *Cypridopsis vidua* (Müller, 1776), 52, dorsal view of *Paranesidea sterreri* Maddocks (1986). 53. Sponge spicules. 54- 55, Branching bryozoans. 56, Cheilostome bryozoan. 57, Bivalve. 58, Brachiopod. 59, Mollusk. 60, Encrusting tube-worm. 61- 63, Teleost (Fish) vertebrae. 64-65, Calcite rafts. All scale bars represent 100 µm.

Table S1| Detailed information on core locations in Palm Cave relative to the Earth's surface. GPS co-ordinates were estimated from overlaying cave survey (≤ 3 m horizontal uncertainty) in Google Earth (± 3 m horizontal uncertainty).

Core Label	Water Depth (m)	Water Depth Uncertainty	Latitude	Longitude	Core termination	Final core length (cm)
SM-C2	20.1	0.3	32.345072°	-64.711072°	sediment	0.78
SM-C3	20.3	0.3	32.345411°	-64.711962°	hardground	0.8
SM-C4	20.7	0.3	32.345627°	-64.711658°	sediment	0.79
SM-C6	20.3	0.3	32.345466°	-64.712108°	sediment	0.78
SM-C7	9.7	0.3	32.345822°	-64.712690°	hardground	0.66
SM-C8	5.3	0.3	32.345869°	-64.712214°	hardground	0.25
SM-C9	20.3	0.3	32.345438°	-64.711141°	hardground	0.57
SM-C10	20.7	0.3	32.344779	-64.711037	hardground	1.75
SM-C11	18	0.3	32.346000°	-64.711450°	sediment	0.46
SM-C12	20.4	0.3	32.345367°	-64.711143°	hardground	0.38
SM-C13	20.4	0.3	32.345417°	-64.711189°	sediment	0.43
SM-C14	20.7	0.3	32.345039°	-64.711257°	hardground	0.88
SM-C15	21.9	0.3	32.345361°	-64.711628°	hardground	0.83

Table S2| Arithmetic averages for measured sedimentary characteristics in the recovered seven different sedimentary units and preserved biological remains. Note that the stable carbon isotopic ratio ($\delta^{13}C_{org}$ ‰ VPBD, analytical uncertainty ±0.2‰) and C:N (analytical uncertainty ±0.1‰) were only determined on cores 3, 6, 10, 14, and 9 (n = 215). Symbols for biological remains: ostracodes (o) and foraminifera (f).

Sediment Group	Sedimentary Unit	Timing of Deposition (Calibrated years before present)	Maximum estimated sedimentation rate	Area of Deposition	Sediment Texture (mg/cc)	Organic Matter Geochemistry			Preserved Biological Remains	Environmental Setting
						Bulk Organic Content (%)	δ13Corg (‰ VPDB)	C:N		
Paleosol	Terra Rosa Paleosol	>11,600		Base of depoists (core 13), expsed in areas with non-deposition (core 8)	66 ± 49 (*n* = 38)	14 ± 1 (*n* = 38)	Not analyzed	Not analyzed	none	Vadose Cave
Organic Deposit	Grey Sapropel	9750 ± 210 to ~9400 (cores 14 and 2)	6 mm/yr (core 2)	Eastern passages only (core 14, core 2)	7 ± 11 (*n* = 89)	16 ± 4 (*n* = 89)	−24.4 ± 0.6‰ (*n* = 33)	17.2 ± 1.0 (*n* = 33)	Darwinula stevensoni (o), Cypridopsis vidua (o)	
Organic Deposit	Dark Brown Sapropel	9620 ± 80 (core 10, 15) to 8520 ± 70 (core 3)	1.6 mm/yr (core 10)	Deepest areas of Palm Cave	4 ± 4 (*n* = 177)	31 ± 8 (*n* = 180)	−24.1 ± 0.9‰ (*n* = 43)	16.8 ± 0.8 (*n* = 43)	Polysaccamina ipohalina (f), Trochammina spp. (f)	Oligohaline Meteoric Lens
Organic Deposit	Light Brown Sapropel	~9180 ± 110 (core 4) to 8370 ± 30 (core 3)	1.25 mm/yr (core 10)	Proximal to Sailors Choice Entrance	9 ± 11 (*n* = 201)	24 ± 4 (*n* = 198)	−23.5 ± 0.8‰ (*n* = 39)	14.9 ± 1.1 (*n* = 39)	Trochammina spp (f)., fish vertebrea	
Iron-rich	Iron and Carbonate	After 8480 ± 60 (core 10)	inadequately constrained	Above limestone, or in deep areas of cave	9 ± 11 (*n* = 21)	15 ± 5 (*n* = 21)	−21.2 ± 2.9‰ (*n* = 22)	13.3 ± 2.9 (*n* = 22)	Bolivina spp. (f),	Mixing Zone
Carbonate	Micrite	By 6600 ± 210, 6600 ± 70 (core 2) to generally ~3000 to 4000 years ago	0.2 mm/yr (c 15)	Widspread throughout Palm Cave	19 ± 33 (*n* = 188)	9 ± 4 (*n* = 188)	−20.4 ± 1.9‰ (*n* = 40)	11.7 ± 2.0 (*n* = 40)	*Spirophthalmidium emaciatum* (f), *Sigmoilina tenuis* (f), *Spirillina vivipara*, *Barbatia domingensis* (b), bryozoans, sponge spicules, brachiopods, serpulid worms	Oxygenated Saline Groundwater Mass
Carbonate	Mixed Carbonate	~3610 ± 480 (core 15)	0.5 mm/yr C7	Widspread throughout Palm Cave	71 ± 117 (*n* = 178	14 ± 1 (*n* = 178)	−19.4 ± 1.2‰ (*n* = 38)	10.3 ± 1.2 (*n* = 38)	*Spirophthalmidium emaciatum* (f), *Sigmoilina tenuis* (f), *Spirillina vivipara*, *Barbatia domingensis* (b), bryozoans, sponge spicules, brachiopods, serpulid worms	

Table S3| Radiocarbon data (*n* = 51) generated by the National Ocean Sciences Accelerator Mass Spectrometry (NOSAMS) facility at Woods Hole Oceanographic Institution, and all were calibrated to sidereal years before present (BP_{1950}) using IntCal 13[5], with the highest probability 2σ result used to frame the results. Other lower probability calibration results do not change the interpretations of the data. Raw calibration data from this table is then rounded to the nearest decade for presentation in Figure 3 in the text, as per radiocarbon convention. The NOSAMS process abbreviations include: (a) gas bench (GB) is the continuous-flow accelerator mass spectrometry technique[6] (CF-AMS), (b) OC is for the standard alkali-acid-alkali organic carbon pretreatment for Standard AMS dating, and (c) HY is for hydrolysis of biogenic carbonate.

Sample Identifier	Core	Depth Mid-Point	Material dated	NOSAMS Process Abbreviation	Sample Processing Type	Accession Number	F Modern	Fm Err	Age	Age Err	δ13C	D13C Source	Highest Probability 2σ Midpoint	Highest Probability 2σ Uncertainty	Probability
SM-C2-14.5 cm	C02	14.5	mollusc	GB	CF-AMS	NA	0.5372	0.0077	4991	115	NA	Not Measured	5650	375	0.949
SM-C2-31.5 cm	C02	31.5	mollusc	GB	CF-AMS	NA	0.4824	0.0071	5855	118	NA	Not Measured	6679	278	1
SM-C2-32.5-33	C02	32.75	mollusc	HY	Standard AMS	OS-112233	0.4861	0.0016	5790	25	1	Measured	6596	66	0.949
SM-C2-47-47.5	C02	47.25	organic carbon	OC	Standard AMS	OS-112146	0.368	0.0014	8030	30	-25.01	Measured	8934.5	79.5	0.719
SM-C2-49.5-50 cm	C02	49.75	organic carbon	OC	Standard AMS	OS-109436	0.3569	0.0014	8280	30	-24.3	Measured	9263	71	0.576
SM-C2-73-74	C02	73.5	Plant/Wood	OC	Standard AMS	OS-112147	0.3448	0.0017	8550	40	-26.48	Measured	9519	34	1
SM-C3_5-6 cm	C03	5.5	mollusc	GB	CF-AMS	OS-129491	0.7645	0.0182	2160	190	NA	Not Measured	2137	411	0.935
SM-C3-15.5 cm	C03	15.5	mollusc	GB	CF-AMS	NA	0.6721	0.0093	3192	112	NA	Not Measured	3395	254	0.987
SM-C3_23-24 cm	C03	23.5	organic carbon	OC	Standard AMS	OS-119838	0.3902	0.0017	7560	35	-22.32	Measured	8374	42	1
SM-C3_59-60 cm	C03	59.5	organic carbon	OC	Standard AMS	OS-119837	0.3816	0.0015	7740	30	-22.44	Measured	8516.5	72.5	1
SM-C3_66-67 cm	C03	66.5	organic carbon	OC	Standard AMS	OS-119837	0.3589	0.0015	8230	35	-24.21	Measured	9189.5	116.5	0.959
SM-C4_24-25 cm	C04	24.5	organic carbon	OC	Standard AMS	OS-119843	0.3836	0.0015	7700	30	-22.53	Measured	8481.5	62.5	1
SM-C4_59-60 cm	C04	59.5	organic carbon	OC	Standard AMS	OS-119844	0.3677	0.0014	8040	30	-25	Measured	8975	46	0.547
SM-C4_72-73 cm	C04	72.5	organic carbon	OC	Standard AMS	OS-119840	0.3596	0.0015	8220	35	-23.97	Measured	9183	111	0.948
SM-C6_10-11 cm	C06	10.5	mollusc	GB	CF-AMS	OS-129493	0.6846	0.0165	3040	190	NA	Not Measured	3205	433	1
SM-C6_19-20 cm	C06	19.5	mollusc	GB	CF-AMS	OS-129494	0.6067	0.0151	4010	200	NA	Not Measured	4436.5	539.5	0.996
SM-C6_20-21 cm	C06	20.5	mollusc	GB	CF-AMS	OS-129495	0.6501	0.016	3460	200	NA	Not Measured	3764.5	526.5	1
SM-C6_70-71 cm	C06	70.5	organic carbon	OC	Standard AMS	OS-119839	0.3576	0.0014	8260	30	-24.11	Measured	9225	97	0.892
SM-C7_10-11 cm	C07	11.5	mollusc	GB	CF-AMS	OS-129497	0.9136	0.0213	725	190	NA	Not Measured	717.5	292.5	0.96
SM-C7_58-60 cm	C07	58.5	mollusc	GB	CF-AMS	OS-129497	0.8621	0.0203	1190	190	NA	Not Measured	1072.5	344.5	0.987
SM-C9_7-8 cm	C09	8.5	mollusc	GB	CF-AMS	OS-129499	0.8809	0.0206	1020	190	NA	Not Measured	976	323	0.99
SM-C9_19-20 cm	C09	19.2	mollusc	GB	CF-AMS	OS-129499	0.6332	0.0156	3670	200	NA	Not Measured	4003	527	0.999
SM-C9_47-48 cm	C09	47.5	organic carbon	OC	Standard AMS	OS-122741	0.3384	0.0016	8700	35	-24.42	Measured	9646.5	99.5	0.993
SM-C10-D1_15.5-16 cm	C10	15.75	organic carbon	OC	Standard AMS	OS-124667	0.3842	0.0014	7690	30	-23.17	Measured	8479	62	1
SM-C10-D2_39-40 cm	C10	109.5	organic carbon	OC	Standard AMS	OS-122742	0.3577	0.0017	8260	35	-23.37	Measured	9228	103	0.86
SM-C10-D2_66-67 cm	C10	136	organic carbon	OC	Standard AMS	OS-122743	0.3493	0.0014	8450	35	-23.62	Measured	9480	47	1
SM-C10-D3_45-46 cm	C10	157	organic carbon	OC	Standard AMS	OS-122744	0.3445	0.0019	8560	45	-24.29	Measured	9535.5	61.5	1
SM-C10-D3_62-63 cm	C10	174.5	organic carbon	OC	Standard AMS	OS-122745	0.3395	0.0015	8680	35	-23.7	Measured	9624	80	0.998
SM-C11_8.5-9 cm	C11	8.75	organic carbon	OC	Standard AMS	OS-122737	0.3567	0.0016	8280	35	-24.37	Measured	9271	135	1
SM-C11_30-31 cm	C11	30.5	organic carbon	OC	Standard AMS	OS-122738	0.3534	0.0016	8360	35	-24.11	Measured	9382	87	1
SM-C12_3-4 cm	C12	4.5	mollusc	GB	CF-AMS	OS-129501	0.7263	0.0174	2570	190	NA	Not Measured	2688	393	0.948
SM-C12_11-12 cm	C12	11.5	mollusc	GB	CF-AMS	OS-129501	0.676	0.0163	3140	190	NA	Not Measured	3298.5	433.5	0.977
SM-C14_31-32 cm	C14	31.5	organic carbon	OC	Standard AMS	OS-135567	0.3383	0.0013	8710	30	-25.88	Measured	9648.5	97.5	0.992
SM-C14_86-87 cm	C14	86.5	organic carbon	OC	Standard AMS	OS-135568	0.3369	0.0013	8740	30	-24.71	Measured	9747.5	207.5	0.881
SM-C15_6-7 cm	C15	6.5	mollusc	GB	CF-AMS	OS-129502	0.8951	0.021	890	190	NA	Not Measured	859	324	0.993
SM-C15_9-10 cm	C15	10.5	mollusc	GB	CF-AMS	OS-129504	0.8478	0.0214	1330	200	NA	Not Measured	1261	365	0.97
SM-C15_10-11 cm	C15	10.5	mollusc	GB	CF-AMS	OS-129504	0.714	0.0173	2710	190	NA	Not Measured	2802.5	452.5	0.987
SM-C15_15-16 cm	C15	15.5	mollusc	GB	CF-AMS	OS-129505	0.7355	0.0175	2470	190	NA	Not Measured	2502.5	458.5	1
SM-C15_19-20 cm	C15	20.5	mollusc	GB	CF-AMS	OS-129507	0.7255	0.0174	2580	190	NA	Not Measured	2691	392	0.951
SM-C15_39-40 cm	C15	39.5	mollusc	GB	CF-AMS	OS-129507	0.6931	0.0167	2940	190	NA	Not Measured	3148.5	426.5	1
SM-C15_40-41 cm	C15	40.5	mollusc	GB	CF-AMS	OS-129508	0.6595	0.016	3340	190	NA	Not Measured	3613.5	475.5	0.991
SM-C15_43-44 cm	C15	43.5	mollusc	GB	CF-AMS	OS-129509	0.633	0.0163	3670	210	NA	Not Measured	4014	556	1
SM-C15_48-49 cm	C15	49.5	mollusc	GB	CF-AMS	OS-129511	0.6481	0.0159	3480	200	NA	Not Measured	3812.5	485.5	0.982
SM-C15_54-55 cm	C15	54.5	mollusc	GB	CF-AMS	OS-129478	0.6264	0.0153	3760	200	NA	Not Measured	4130	517	0.981
SM-C15_63-64 cm	C15	64.5	mollusc	GB	CF-AMS	OS-129480	0.5941	0.015	4180	200	NA	Not Measured	4761.5	538.5	0.986
SM-C15_64-65 cm	C15	64.5	mollusc	GB	CF-AMS	OS-129480	0.6087	0.0151	3990	200	NA	Not Measured	4427.5	541.5	1
SM-C15_65-66 cm	C15	66.5	mollusc	GB	CF-AMS	OS-129482	0.5243	0.0136	5190	210	NA	Not Measured	5991.5	412.5	0.987
SM-C15_66.5 cm	C15	67.5	mollusc	HY	Standard AMS	OS-129619	0.5938	0.0015	4190	20	1.23	Measured	4726	36	0.595
SM-C15_71 cm	C15	71	Plant/Wood	OC	Standard AMS	OS-129934	0.3616	0.0014	8170	30	NA	Not Measured	9081.5	63.5	0.793
SM-C15_79-80 cm	C15	79.5	organic carbon	OC	Standard AMS	OS-122740	0.339	0.0019	8690	45	-27.51	Measured	9661.5	121.5	0.995
SM-C15_79-80 cm	C15	79.5	organic carbon	OC	Standard AMS	OS-129829	0.3383	0.0015	8710	35	-26.81	Measured	9665	117	0.996

Table S4| Different in mineralogy between the terra rosa paleosols and iron-rich sedimentary units. Some samples were re-analyzed after treatment with 10% HCl to observe remaining minerals after removal of carbonates (aragonite and calcite), and amplify the XRD-signal of the non-acidified mineral residue.

Core	Depth Interval	Sedimentary unit	Pretreatment	Minerals (Relative Abundance %)											
				Crandallite	Kaolinite	Quartz	Goethite	Pyrite	Aragonite	Calcite	Woodhouseite	Maghemite	Lepidocrocite	Rectorite	Montroseite
8	5-6		none	43.5	12.2	39.1	5.2	0	0	0	0	0	0	0	0
8	10-11		none	50.3	14.7	28.8	6.3	0	0	0	0	0	0	0	0
8	15-16	terra rosa paleosol	none	58	17.7	16.6	7.7	0	0	0	0	0	0	0	0
13	35-36		none	50	17.4	20.5	12.1	0	0	0	0	0	0	0	0
13	40-41		none	61	20.6	9.3	9	0	0	0	0	0	0	0	0
			Arithmetic averages	*52.6*	*16.5*	*22.9*	*8.1*	0	0	0	0	0	0	0	0
14	14		none	0	0	0	0	0	53.3	36.4	9.4	0	0.9	0	0
14	14		10% HCl	0	0	87.5	2.8	0	0	0	6.2	0	0	2.4	1.1
15	15		none	25.8	17.3	8.2	30.3	18.4	0	0	0	0	0	0	0
4	7		none	0	0	0	0	0	68.4	31.6	0	0	0	0	0
4	7		10% HCl	0	17.2	22.3	0	0	8.1	1.2	39.9	9.2	2.1	0	0
4	16	iron and carbonate	none	0	16.7	28.4	20.7	0	0	26.8	7.4	0	0	0	0
4	16		10% HCl	0	23.1	10.8	37.2	0	0	2	26.9	0	0	0	0
10	13		none	0	0	67.8	3.7	0	0	27.8	0	0	0.7	0	0
10	13		10% HCl	0	4.4	82.6	6.9	0	0	0	5	0	1	0	0
10	145		none	0	0	61.6	2.9	0	0	27.9	4.4	0	3.2	0	0
10	145		10% HCl	0	0	87.7	3.9	0	0	0.4	4.8	0	3.2	0	0
			Arithmetic averages	5.16	5.666667	27.6667	9.6	3.0667	20.2833	25.0833	3.533333333	0	0.8	0	0

References

1 Patterson, R. T. & Fishbein, E. Re-examiniation of the statistical methods used to determine the number of point counts needed for micropaleontological quantitative research. *J. Paleontol.* **63**, 245-248 (1989).

2 Carmen, K. W. *The shallow-water foraminifera of Bermuda* PhD thesis, Massachusetts Institute of Technology, (1927).

3 van Hengstum, P. J. & Scott, D. B. Ecology of foraminifera and habitat variability in an underwater cave: distinguishing anchialine versus submarine cave environments. *J. Foram. Res.* **41**, 201-229 (2011).

4 Loeblich, A. R., Jr., & Tappan, H. *Foraminiferal genera and their classification*. Vol. 2 vols (Van Nostrand Reinhold Company, 1987).

5 Reimer, P. J. *et al.* IntCal13 and Marine13 radiocarbon age calibration curves 0-50,000 years Cal BP. *Radiocarbon* **55**, 1869-1887 (2013).

6 McIntyre, C. P. *et al.* Rapid radiocarbon (^{14}C) analysis of coral and carbonate samples using a continuous-flow accelerator mass spectrometry system. *Paleoceanography* **26**, 6 (2011).
